# Ferroptosis-Related Long Non-Coding RNA signature predicts the prognosis of Head and neck squamous cell carcinoma

**DOI:** 10.7150/ijbs.55552

**Published:** 2021-01-31

**Authors:** Yun Tang, Cheng Li, You-Jing Zhang, Zeng-Hong Wu

**Affiliations:** 1 Department of Critical Care Medicine, Union Hospital, Tongji Medical College, Huazhong University of Science and Technology, Wuhan, 430022, China.; 2 Department of Otolaryngology Head and Neck Surgery, The Central Hospital of Wuhan, Tongji Medical College Huazhong University of Science and Technology, Wuhan, Hubei, China.; 3 School of Public Health, Tongji Medical College, Huazhong University of Science and technology, Wuhan, China.; 4 Department of Otorhinolaryngology, Union Hospital, Tongji Medical College, Huazhong University of Science and Technology, Wuhan, Hubei, China.; 5 Department of Infectious Diseases, Union Hospital, Tongji Medical College, Huazhong University of Science and Technology, Wuhan 430022, China.

**Keywords:** Head and neck squamous cell carcinoma, ferroptosis, genes, lncRNAs, immune infiltration, data mining.

## Abstract

**Background**: Head and neck squamous cell carcinoma (HNSCC) are head and neck cancers. On the other hand, ferroptosis is a novel iron-dependent and ROS reliant type of cell death observed various disease conditions.

**Method**: We constructed a prognostic multilncRNA signature based on ferroptosis-related differentially expressed lncRNAs in HNSCC.

**Results**: We identified 25 differently expressed lncRNAs associated with prognosis of HNSCC. Kaplan-Meier analyses revealed the high-risk lncRNAs signature associated with poor prognosis of HNSCC. Moreover, the AUC of the lncRNAs signature was 0.782, underscoring their utility in prediction HNSCC prognosis. Indeed, our risk assessment model was superior to traditional clinicopathological features in predicting HNSCC prognosis. GSEA revealed the immune and tumor-related pathways in the low risk group individuals. Moreover, TCGA revealed T cell functions including cytolytic activity, HLA, regulation of inflammationp, co-stimulation, co-inhibition and coordination of type II INF response were significantly different between the low-risk and high-risk groups. Immune checkpoints such as PDCD-1 (PD-1), CTLA4 and LAG3, were also expressed differently between the two risk groups.

**Conclusion:** A novel ferroptosis-related lncRNAs signature impacts on the prognosis of HNSCC.

## Introduction

Head and neck squamous cell carcinoma (HNSCC) are neoplasms affecting different tissues and organs in the head and neck region including the tongue, mouth, nasopharynx, larynx and throat 1. Every year, there are over 655,000 new HNSCC cases (globally), resulting in 90,000 deaths 2. Tobacco smoking, alcohol consumption and human papillomavirus (HPV) infection are important risk factors for developing HNSCC 3. Moreover, Epstein-Barr virus (EBV) is strongly associated with nasopharyngeal carcinoma (NPC) 4. Although several treatment options such as surgery, radiotherapy and chemotherapy are available, the asymptomatic nature and lack of early detection of HNSCC result in less than 50% 5-year survival rate of individuals with the cancers. In addition, due to the same reasons, even after treatment, the risk of recurrence and metastasis of HNSCC remain high 5, 6. Efficient clinical management of HNSCC has been constrained by suboptimal preclinical models or lack of accurate biomarkers for early diagnosis of the cancers. Currently, there are several anti-tumor drugs that induce apoptosis of cancer cells 7. Thus, it is particularly critical to explore other forms of cell death to overcome tumor cell resistance while at the same time uncovering new and efficient prognostic biomarkers for HNSCC.

Past decades have seen a rapid increase in research on tumor ferroptosis. This is an iron-dependent cell death reliant on intracellular accumulation of reactive oxygen species (ROS), distinct from apoptosis and autophagy 8. Dysregulation of iron metabolism is a risk factor for cancer and as well promotes tumor growth. Compared with normal cells, cancer cells exhibit iron addiction, which is the over dependence on iron for proliferation 9. Indeed, activation of ferroptosis pathways may override drug resistance experienced by current chemotherapeutic agents, opening a new therapeutic frontier for cancer treatment. Meanwhile, long non-coding RNAs (lncRNA) are a subset of RNA molecule about 200 nt, which regulate the expression of genes 10. Apart from gene regulation, lncRNA participates in various biological regulatory processes, including those implicated for occurrence, development and metastasis of tumors 11. At the moment, there are very few studies on ferroptosis-related lncRNAs. One recent study revealed that lncRNA LINC00618 is under-expressed in human leukemia cells. The mRNA promotes ferroptosis by increasing the levels of lipid ROS and iron, while decreasing the expression of *SLC7A11*. It further induces vincristine-induced ferroptosis and apoptosis of 12. In a related study, Lu* et al.* 13 found that in acute ischemic stroke, lncRNA PVT1 regulated ferroptosis via miR-214, which modulates the expression of *TFR1* and *TP53*. Similarly, silencing lncRNA ZFAS1 attenuated ferroptosis and significantly modulated inflammation and lipid peroxidation 14. However, sequence-based studies that systematically evaluate ferroptosis-related lncRNA signature and its association with overall survival (OS) in HNSCC patients remain scanty. In this study, we first constructed a prognostic multi-lncRNA signature of differentially expressed ferroptosis-related lncRNA based on the Cancer Genome Atlas (TCGA) data. We then explored the role of ferroptosis-related mRNA, N6-methyladenosine (m6A) mRNA statues and immune responses in HNSCC prognosis.

## Methods

### Data collection

RNA-sequence (42 normal and 487 tumor) data of 511 patients was extracted from the TCGA-HNSCC database. The clinical characteristics of the patients are shown in **Table 1**. The corresponding ferroptosis-related genes were downloaded from FerrDb 15, a web-based consortium that provided a comprehensive and up-to-date database for ferroptosis markers, their regulatory molecules and associated diseases. Overall, we identified 259 (Driver: 108; suppressor: 69; marker: 111) ferroptosis-related genes (**Table S1**). The relationship between the ferroptosis-related lncRNAs and HNSCC was assessed using Pearson correlation. The association was considered significant if the correlation coefficient |*R^2^*|>0.3 at *P*<0.001. The collected clinical-pathological data of the HNSCC patients included gender, age, stage, grade, TMN, survival status and survival time. Significant differential expression of ferroptosis-related lncRNAs was set at FDR<0.05 and |log_2_FC|≥1. First, we explored the function of both upregulated and downregulated ferroptosis-related differentially expressed genes (DEGs). We then used Gene ontology (GO) to evaluate the biological pathways associated with the DEGs. Further function analysis of biological processes (BP), molecular functions (MF) and cellular components (CC)) regulated by the differently expressed ferroptosis-related lncRNAs were analyzed based on Kyoto Encyclopedia of Genes and Genomes (KEGG) data using R software, ggplot2 package.

### Development of the ferroptosis-related lncRNAs prognostic signature

We used Lasso‐penalized Cox regression and Univariate Cox regression analyses to construct the ferroptosis-related lncRNAs signature, stratified based on risk score (Coefficient lncRNA1 × expression of lncRNA1) + (Coefficient lncRNA2 × expression of lncRNA2) + ⋯ + (Coefficient lncRNAn × expression lncRNAn). The associated risk score for each HNSCC patients were also evaluated. The RNAs were classified in either low-risk (<median number) or high-risk (≥ median number) group based on the median score.

### The predictive nomogram

We performed Gene set enrichment analyses (GSEA) to define the lncRNAs signatures in the KEGG, which were then searched in the TCGA-HNSCC database. Statistical significance was set at *P*<0.05 and false discovery rate (FDR) q<0.25. A nomogram was constructed integrating the prognostic signatures, for predictive of 1, 3, and 5‐year OS of HNSCC patients.

### Immunity analysis and gene expression

At the same time, the CIBERSORT 16, 17, ESTIMATE 18, MCPcounter 19, single-sample gene set enrichment analysis (ssGSEA) 20 and TIMER 21 algorithms were compared to assess cellular components or cell immune responses between high risk and low risk group based on ferroptosis-related lncRNAs signature. The differences in immune response under different algorithms were uncovered using a Heatmap. In addition, ssGSEA was used to quantify the tumor-infiltrating immune cell subgroups between the two groups as well as assessing their immune function. Potential immune checkpoint was also retrieved from previous literature.

### Statistical analysis

Data was analyzed using Bioconductor packages in R software, version 4.0.2. Normally and non-normally distributed variables were analyzed using the unpaired student's t-test and the Wilcoxon test, respectively. Benjamini-Hochberg method was used to identify the differently expressed lncRNAs, based on FDR. The ssGSEA-normalized HNSCC DEGs were compared with a genome using "GSVA" (R-package). The sensitivity and specificity of the derive prognostic signatures for HNSCC in comparison to other clinicopathological was assessed using the operating characteristic curve (ROC) and decision curve analysis (DCA) 22. The relationship between ferroptosis-related lncRNAs and clinicopathological manifestations was evaluated using logistic regression analyses and a heatmap graph. The survival analysis of HNSCC patients based on the ferroptosis-related lncRNAs signature was assessed using the Kaplan-Meier survival analysis. For each analysis, statistical significance was set at *P* <0.05.

## Results

### Enrichment Analysis of ferroptosis-related genes

**Figure 1:**
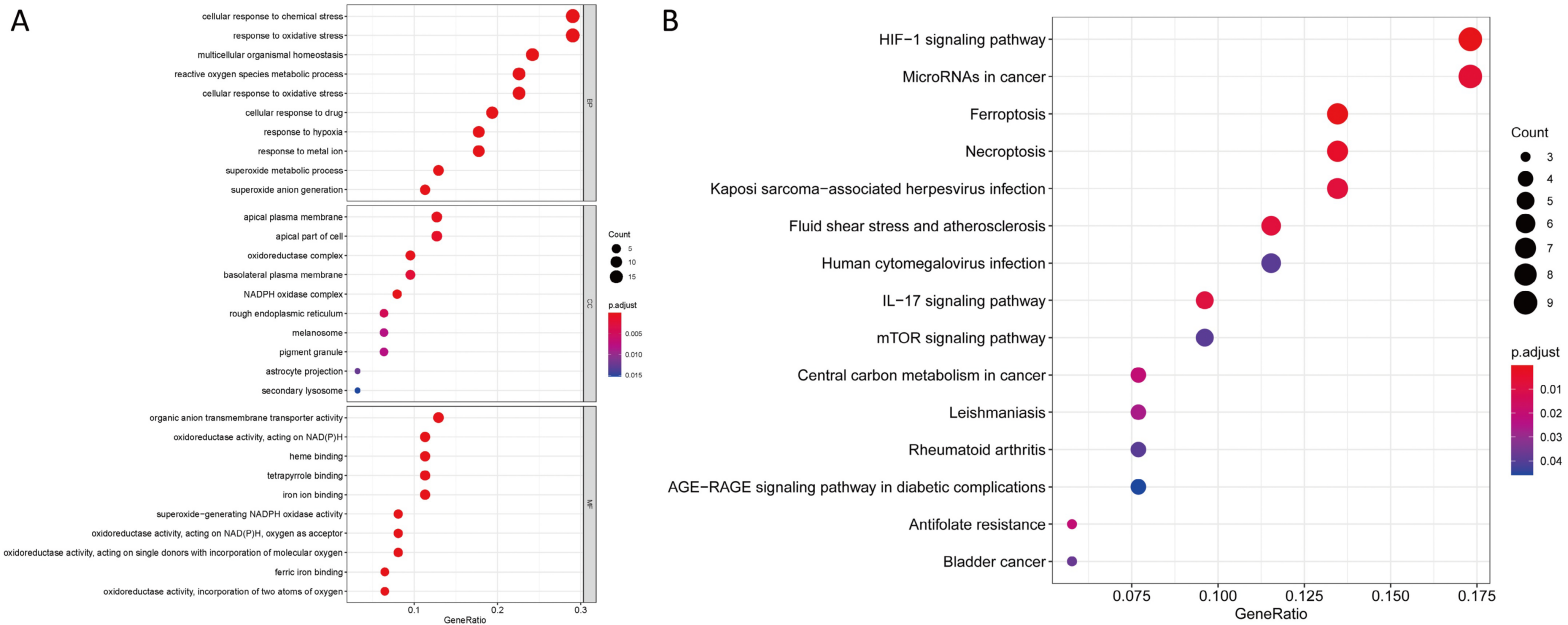
GO and KEGG analyses for ferroptosis-related differentially expressed genes. **(A)** GO and **(B)** KEGG.

We uncovered 65 ferroptosis-related DEGs (18 downregulated and 47 upregulated; **Table S2**). BP participated in the production of oxidative stress superoxide anion, homeostasis and metabolism of superoxides among others. MF mainly regulated production of nicotinamide adenine dinucleotide phosphate (NADPH) oxidase, molecular oxygen and binding of ferric iron.CC were mainly up-regulated in NADPH oxidase complex, oxidoreductase complex and apical plasma membrane synthesis pathways. KEGG based analysis revealed the over-expressed genes were mainly involved in ferroptosis, hypoxia-inducible factor (HIF)-1 signaling pathway, synthesis of microRNAs in cancer cells, IL-17 signaling pathway, central carbon metabolism in cancer cells, bladder cancer, human cytomegalovirus infection and mTOR signaling pathway (**Figure 1** and **Table S3**).

### The ferroptosis-based lncRNAs prognostic signature

We uncovered 935 ferroptosis-related lncRNAs (**Table S4**). Univariate COX analysis identified 75 significant ferroptosis-related lncRNAs, which were included in the multivariate COX analysis. Overall, 25 differently expressed lncRNAs (LINC01963, AL357033.4, LINC01980, AL132989.1, AATBC, ELF3-AS1, AC135050.6, AL161431.1, AC106820.3, AL451085.2, AC007991.2, AC136475.2, AC012467.2, AC144831.1, AC116914.2, AC008115.3, PSMA3-AS1, PCED1B-AS1, AL139158.2, EP300-AS1, AC104083.1, AL022328.2, AC012640.2, PAX8-AS1, and AL450992.2) were found to be independent prognosis predictors of HNSCC (**Table S5**). Thus, we calculated risk scores and constructed a prognostic signature for the lncRNAs.

### Survival results and multivariate examination

**Figure 2:**
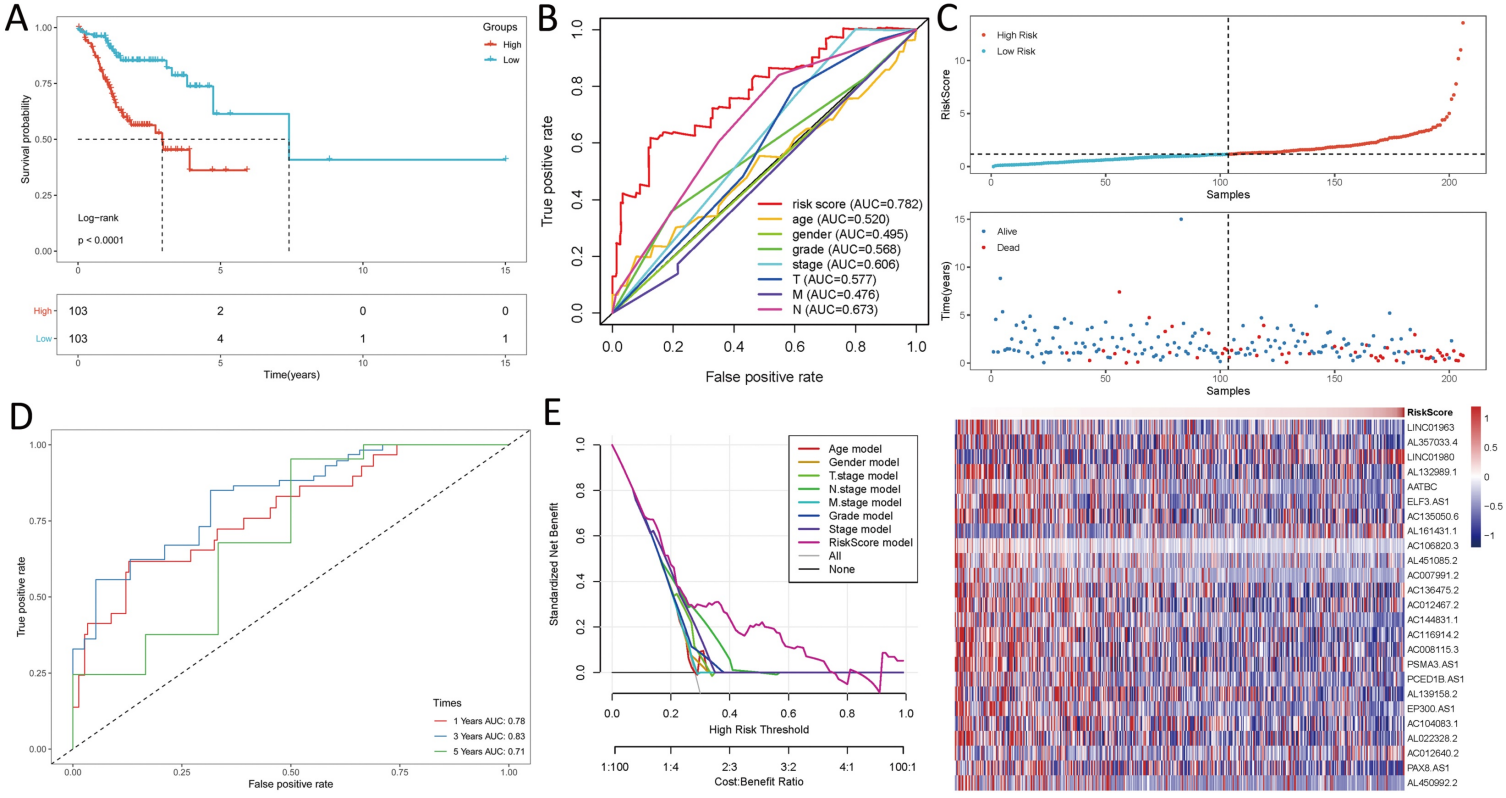
Ferroptosis-related lncRNAs signature based on TCGA. **(A)** Kaplan-Meier curves result, **(B)**. The AUC values of the risk factors, **(C)**. Risk survival status plot, **(D)** The AUC of the for the prediction of 1, 3, 5-year survival rate of HNSCC and **(E)** The DCA of the risk factors.

**Figure 3:**
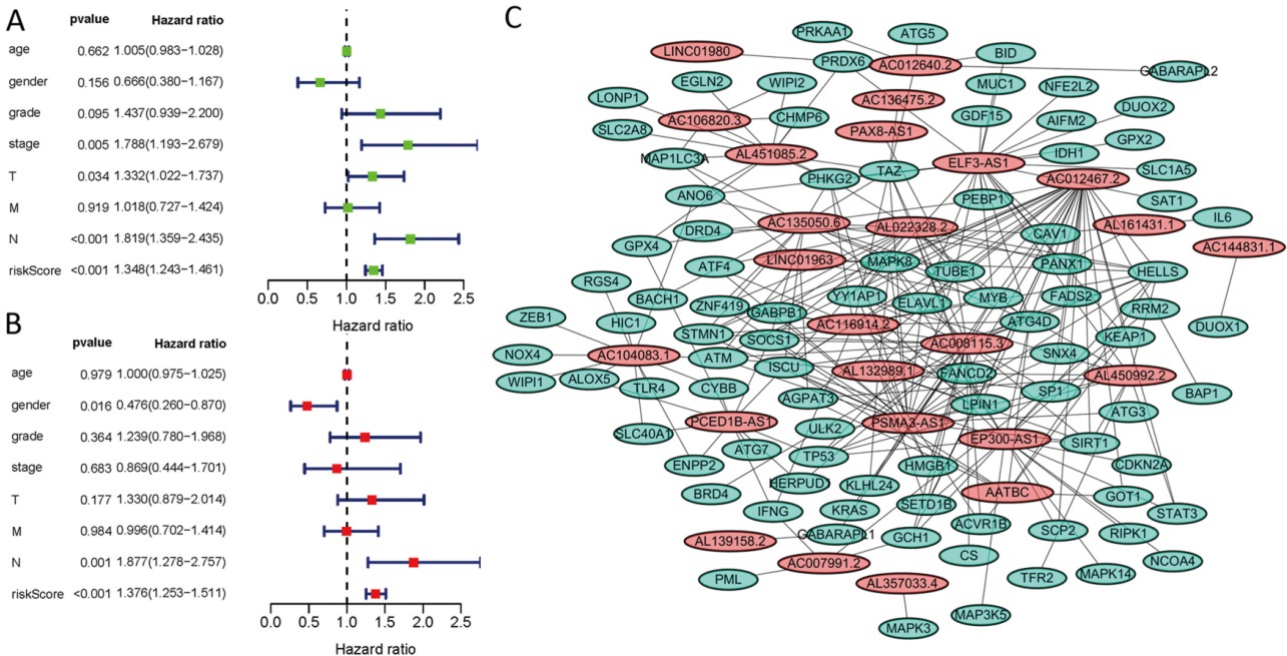
Univariate and multivariate COX analysis for the expression of ferroptosis-related lncRNAs. **(A).** univariate, **(B).** multivariate, **(C).** The relationship between the novel lncRNA and mRNA expression.

**Figure 4:**
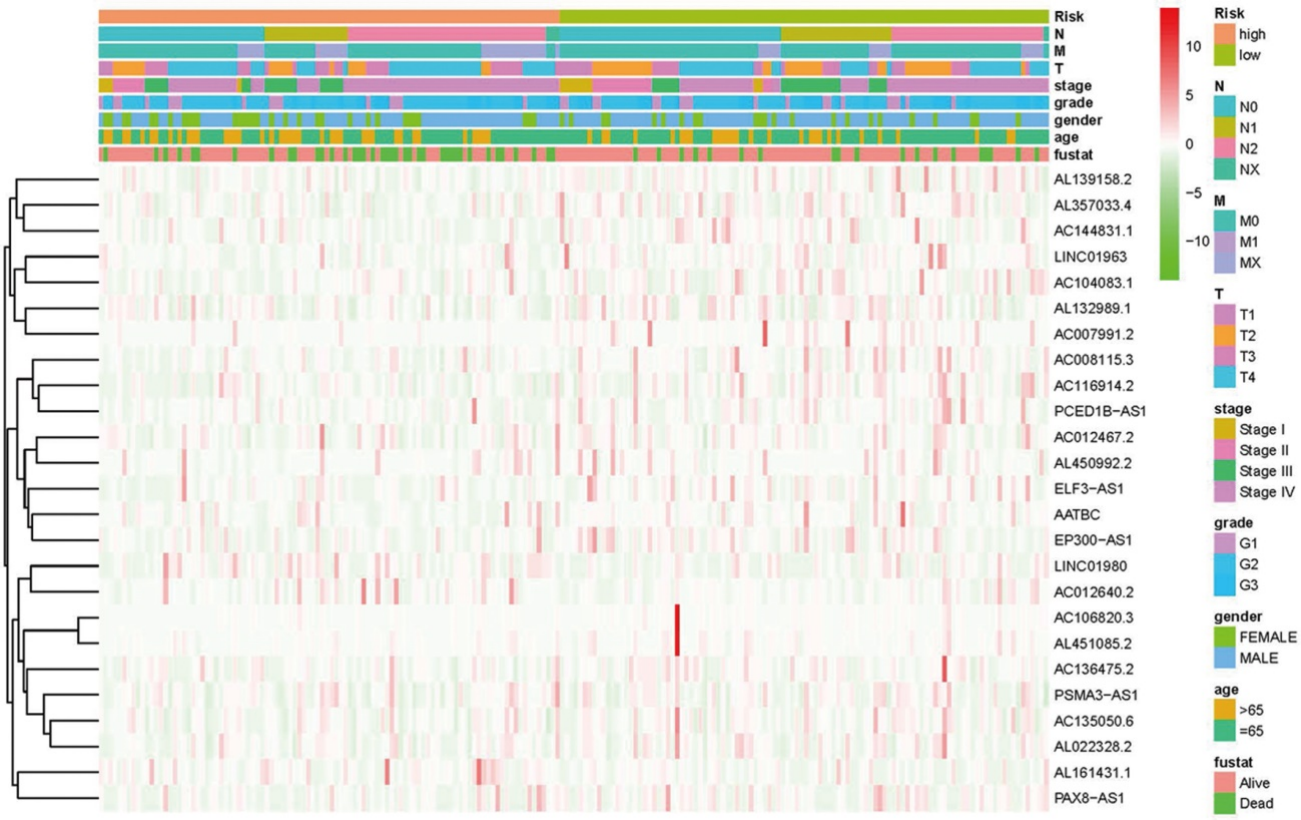
Heatmap for ferroptosis-related lncRNAs prognostic signature and clinicopathological manifestations.

**Figure 5:**
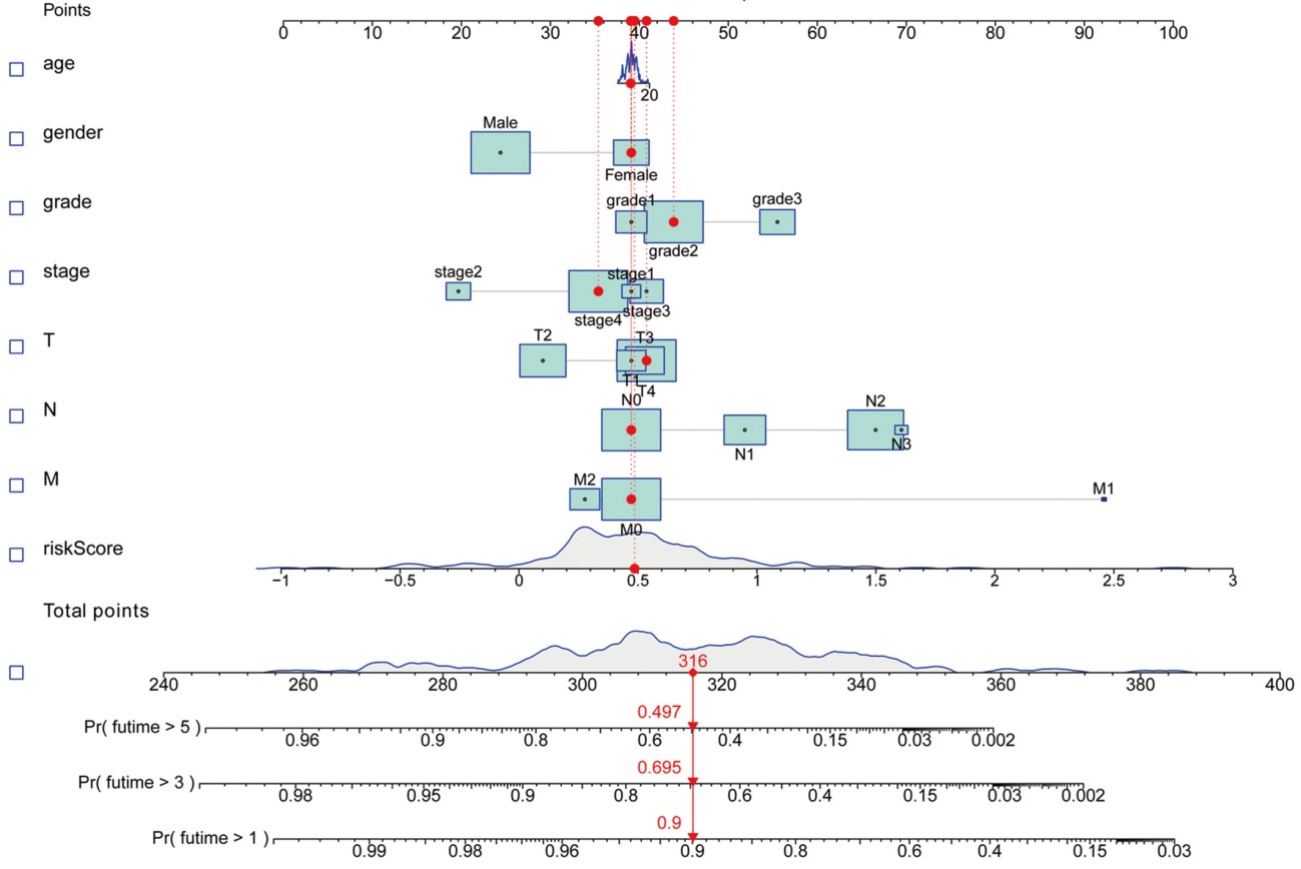
A nomogram for both clinic-pathological factors and prognostic ferroptosis-related lncRNAs.

Kaplan-Meier analyses revealed the expression of high-risk lncRNAs signatures corresponded with poorer survival (*P*<0.001, **Figure 2A**). Meanwhile, the AUC of the signature lncRNAs was 0.782, exhibiting superior performance than the traditional clinicopathological features in predicting the prognosis of HNSCC (**Figure 2B, 2E**). Using patient's risk survival status plot, we found the patient's risk score was inversely proportional to the survival of patients with HNSCC. Interestingly, our heatmap demonstrated that most of the novel lncRNAs uncovered in this study were negative correlation with our risk model and needs more researches to conduct (**Figure 2C**). The AUC predictive value of the novel IncRNAs signature for 1, 3, 5-year survival rate was 0.78, 0.83 and 0.71, respectively (**Figures 2D**). Univariate and multivariate COX analysis revealed that IncRNAs signature (HR: 1.38, 95CI: 1.25-1.51) as well as tumor stage (HR: 1.88, 95CI: 1.28-2.76) were independent prognosis factors of OS of HNSCC patients (**Figure 3A, 3B**). The relationship between lncRNA and mRNA is shown in **Figure 3C**. The heatmap for the association between ferroptosis-related lncRNAs prognostic signature and clinicopathological manifestations were also analyzed (**Figure 4**). The hybrid nomogram incorporating clinicopathological characteristics and the novel ferroptosis-related lncRNAs prognostic signature (**Figure 5**) was stable and accurate, thus may be applied in clinical management of HNSCC patients.

### Gene set enrichment analyses

**Figure 6:**
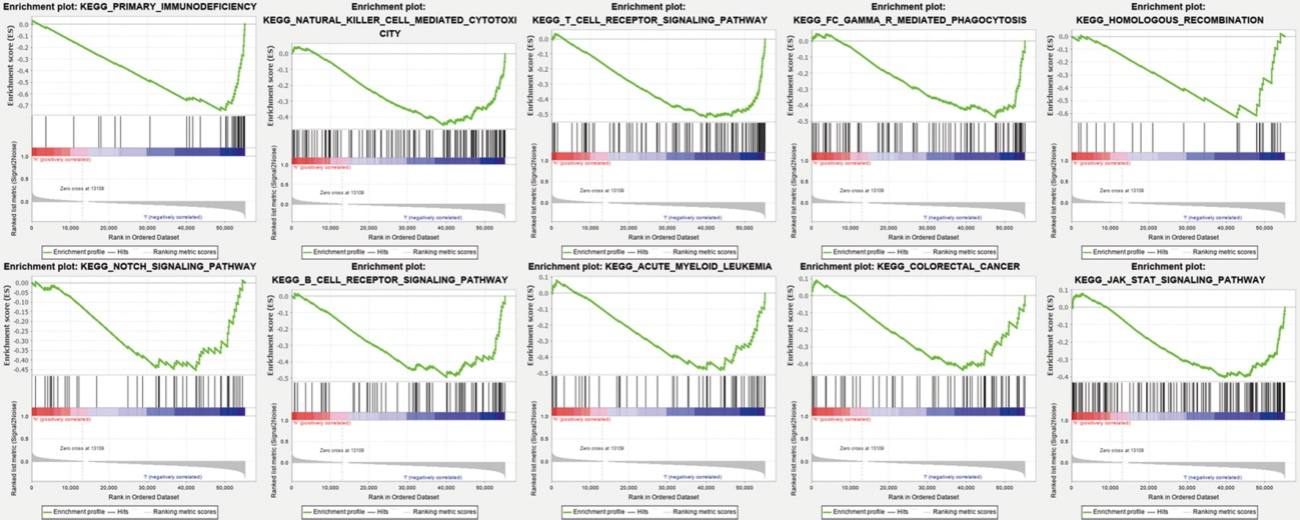
Gene enrichment analysis for ferroptosis-related lncRNAs based on TCGA.

Gene set enrichment analyses (GSEA) revealed the majority of the novel ferroptosis-related lncRNAs prognostic signature regulated immune and tumor-related pathways such as primary immunodeficiency, natural killer cell mediated cytotoxicity, T/B cell receptor signaling pathway, FC gamma R mediated phagocytosis, homologous recombination, notch signaling pathway, acute myeloid leukemia, colorectal cancer, and jak stat signaling pathway (**Figure 6**) and (**Table S6**).

### Immunity and gene expression

**Figure 7:**
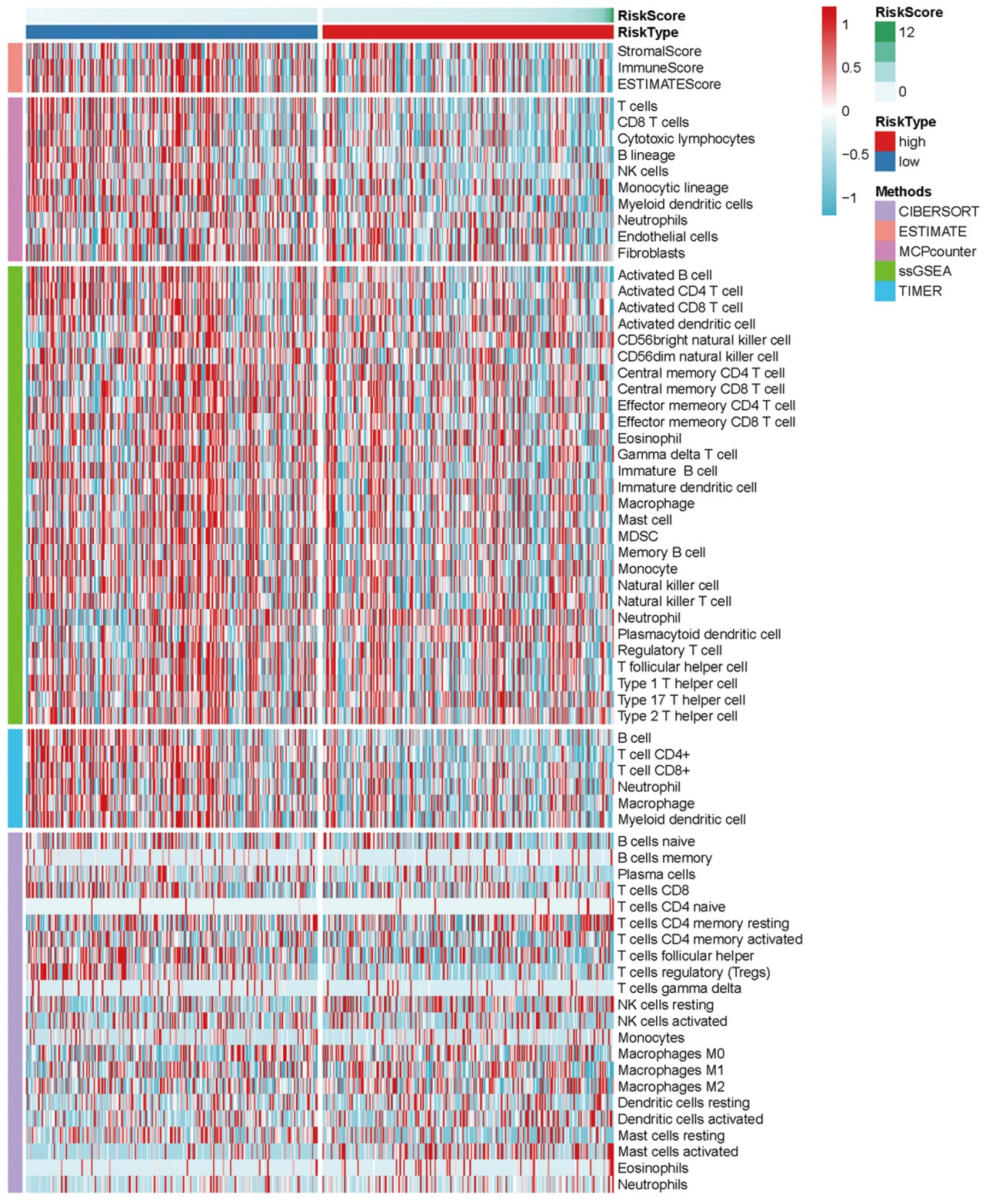
Heatmap for immune responses based on CIBERSORT, ESTIMATE, MCPcounter, ssGSEA, and TIMER algorithms among high and low risk group.

**Figure 8:**
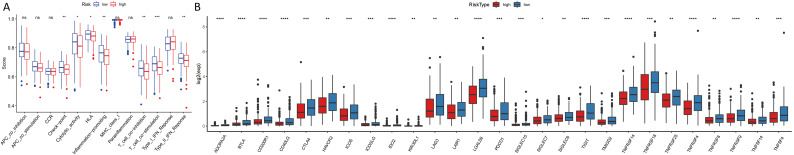
** (A).** ssGSEA for the association between immune cell subpopulations and related functions **(B).** Expression of immune checkpoints among high and low HNSCC risk groups.

**Figure 9:**
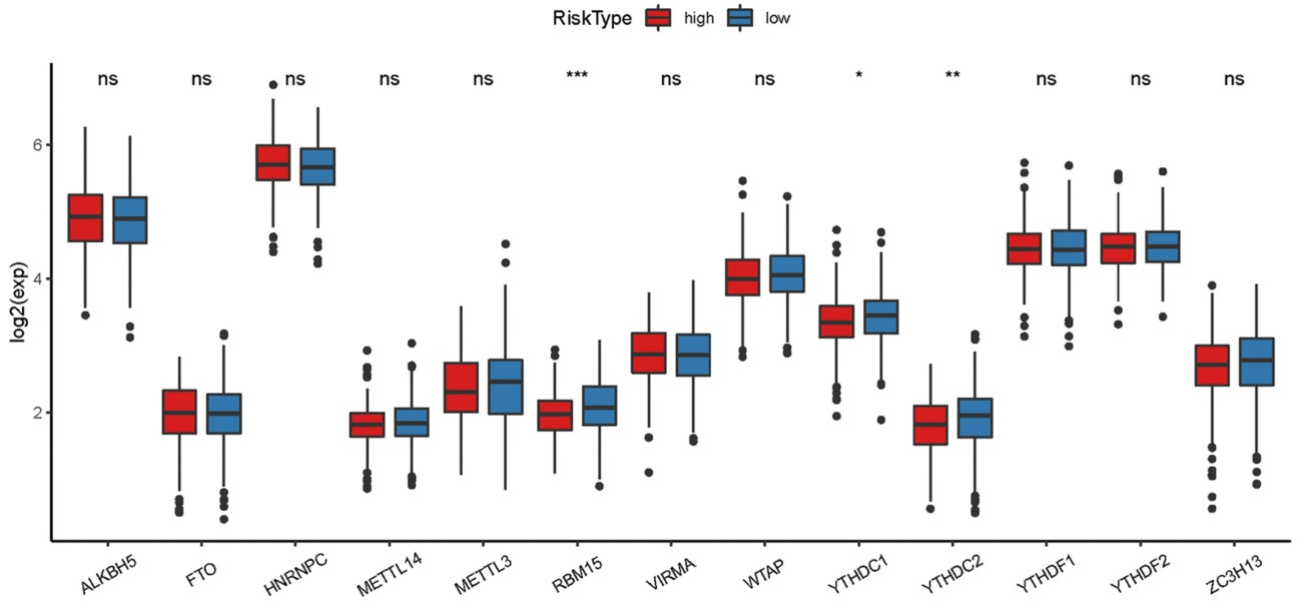
The expression of m6A-related genes between high and low HNSCC risk group.

The heatmap of immune responses based on CIBERSORT, ESTIMATE, MCP counter, single-sample gene set enrichment analysis (ssGSEA) and TIMER algorithms is shown in **Figure 7**. Correlation analysis between immune cell subpopulations and related functions based on ssGSEA of TCGA-HNSCC data revealed that T cell functions including checkpoint (inhibition), cytolytic, HLA, regulation of inflammation, co-stimulation of, co-inhibition of and type II INF response were significantly different between the low-risk and high-risk groups **Figure 8A**. Given the importance of checkpoint inhibitor-based immunotherapies, we further explored the difference in the expression of immune checkpoints between the two groups. We found a substantial difference in the expression of PDCD-1 (PD-1), CTLA4, LAG3, BTLA among others, between the two groups of patients (**Figure 8B**). The comparison in the expression of m6A-related mRNA expression between high and low risk group suggested that the expression of* RBM15*,* YTHDC1*, and *TDHDC2* were significance (**Figure 9**).

## Discussion

Ferroptosis can overcome resistance of malignant cells to chemotherapy and as well facilitate removal of defective cells. As such, it's potentially a novel approach for tumor treatment. In this study, we first identified novel ferroptosis-related prognostic lncRNAs signature based on the TCGA dataset. We then explored the roles of immune infiltrating cells in tumor microenvironment and immune checkpoint inhibitors in the prognosis of HNSCC. Findings of this study uncovered potential biomarker and therapeutic target in the ferroptosis signaling pathways.

Overall, out analyses uncovered 65 ferroptosis-related DEGs. KEGG analyses further revealed the genes mainly participated in hypoxia-inducible factor (HIF)-1 signaling pathway, expression of cancer related microRNAs, IL-17 signaling pathway, central carbon metabolism in cancer cells and mTOR signaling pathway. A recent study found that FG-4592 (an inhibitor of prolyl hydroxylase of HIF) pretreatment reduces ferroptosis in advanced stage of kidney injury by activating Nrf2 via the Akt/GSK-3β-mediated 24. Takahashi *et al.* 25 reported that cancer spheroids utilize the mammalian target of rapamycin (mTOR) for proliferation and the lipid peroxidase *GPX4* for defense against ferroptosis. Overall, in this study, 25 differently expressed lncRNAs were found to be independent prognosis factors for HNSCC. A recent study found over-expression of LINC01963 in pancreatic carcinoma cells inhibited proliferation of pancretic cells via the miR-641/TMEFF2 pathway 26. Up-regulated expression of LINC01980 in esophageal squamous cell carcinoma confers poor prognosis and as well promotes epithelial-mesenchymal transition (EMT) via the miR-190a-5p and MYO5A pathways 27. In a related study, lncRNA AATBC was found to be over-expressed in NPC, corresponding to poor survival. Over-expression of lncRNA AATBC also promoted migration and invasion of NPC cells, mediated by miR-1237-3p 28. lncRNA ELF3-antisense RNA 1 (ELF3-AS1) promotes the proliferation of oral squamous cell carcinoma (OSCC) cells by disrupting glucose metabolism. Overall, over-expression of ELF3-AS1 conferred poor OS of OSCC patients 29, 30. Similarly, one recent study reported that AL161431.1 target and binds miR-1252-5p, inhibiting MAPK signaling in endometrial carcinoma cells 31. PSMA3-AS1 knockdown suppresses the migration, proliferation and invasion of glioma cells *in vivo* and *in vitro* and represses proteasome inhibitors 32, 33. Expression of PCED1B-AS1, closely associated with larger tumor size, higher grade, poor survival, rapid proliferation, glucose uptake and lactate release in cancer cells, was found to be upregulated in glioblastoma cells and tissues 34. Paired box 8 antisense RNA 1 (PAX8-AS1) polymorphisms rs4848320 and rs6726151 are risk factor for the development of childhood acute lymphoblastic leukemia, but decreases the risk of developing cervical cancer 35, 36. On the other hand, one study reported thatdown-regulation of AL450992.2 was associated with autoimmune disorders 37. However, to date, there is no study on the role of ferroptosis-associated lncRNAs in the prognosis of HNSCC. Such findings may provide invaluable sight into the future control of cancers.

Here, the differently expressed ferroptosis-associated lncRNAs were stratified into two categories of high‐ and low‐risk to explore their potential roles in HNSCC. Ferroptosis combined with immune checkpoint inhibitors (ICIs) synergistically enhance antitumor activity, even in ICI-resistant types 38. There are currently very few studies exploring the relationship between ICI and ferroptosis. Increasing evidence suggests that microRNA (miRNA) and lncRNA are critical in mediating the regulation of ferroptosis. Nrf2 inhibits iron absorption, reducing production of ROS. As such, miRNA disrupt ferroptosis by regulating the expression of Nrf2 39-41. Meanwhile, miRNA participates in regulating transport, storage, utilization and absorption of iron. In recent years, new ferroptosis regulatory factors such as P53, ATF3/4, SLC7A11, ACSL4 and BECN1 pathway have been discovered. Intriguingly, lncRNA plays a critical role in regulating the expression of these factors 42.

Ferroptosis is a new form of cell death that can potentially provide a new approach in tumor treatment. However, many key issues such as the interconnection between ferroptosis and other cell deaths and host immunogenicity remain unsolved. Thus, this study explored ferroptosis biomarkers useful in predicting the prognosis of HNSCC, which can inform treatment modalities for the diseases. Nonetheless, our signature profiles need further validation using different cohorts. Given that our findings were not validated using clinical samples, the reliability of our results cannot be fully guaranteed. Also, based on the limited clinical data, our findings should be applied with caution. In general, the prognostic prediction model developed in this study needs further validation.

## Conclusion

Specific ferroptosis associated lncRNAs can predict the prognosis of HNSCC.

## Supplementary Material

Supplementary tables.

## Authors' contributions

W.Z.H. designed and analyzed the research study; W.Z.H. wrote and revised the manuscript, T.Y.; L.C. and Z.Y.J. collected and analysis the data and all authors have read and approved the manuscript.

## Figures and Tables

**Table 1:** The clinical characteristics of patients in the TCGA dataset.

Variable	Number of samples
Gender	
Male/Female	374/137
Age at diagnosis	
≤65/>65/NA	336/174/1
Grade	
G1/G2/G3/G4/NA	60/303/118/7/23
Stage	
I/II/III/IV/NA	27/75/81/256/72
T	
T0/T1/T2/T3/T4/NA	1/49/140/99/161/61
M	
M0/M1/NA	184/1/326
N	
N0/N1/N2/N3/NA	172/67/167/8/97

## Abbreviations

HNSCC: Head and neck squamous cell carcinoma
ROS: reactive oxygen species
miRNA: microRNA
OS: overall survival (OS)
DEGs: differentially expressed genes
TCGA: The Cancer Genome Atlas
GO: Gene ontology
BP: biological processes
MF: molecular function
CC: cellular components
KEGG: Kyoto Encyclopedia of Genes and Genomes
GSEA: Gene set enrichment analyses
FDR: false discovery rate
ssGSEA: single-sample gene set enrichment analysis (ssGSEA)
